# Long-Term Effect of Therapeutic Horseback Riding in Youth With Autism Spectrum Disorder: A Randomized Trial

**DOI:** 10.3389/fvets.2018.00156

**Published:** 2018-07-16

**Authors:** Robin L. Gabriels, Zhaoxing Pan, Noémie A. Guérin, Briar Dechant, Gary Mesibov

**Affiliations:** ^1^University of Colorado Anschutz Medical Campus, Aurora, CO, United States; ^2^Children's Hospital Colorado, Aurora, CO, United States; ^3^Department of Comparative Pathobiology, Center for the Human-Animal Bond, College of Veterinary Medicine, Purdue University, West Lafayette, IN, United States; ^4^Frank Porter Graham Child Development Institute, University of North Carolina, Chapel Hill, NC, United States

**Keywords:** animal-assisted interventions, autism spectrum, therapeutic horseback riding, long-term outcomes, irritability

## Abstract

This paper presents 6-month follow-up data of 44% (*N* = 64/116) of participants (ages 6–16 years) with a diagnosis of autism spectrum disorder, who participated in a previously-published randomized controlled trial of therapeutic horseback riding (THR) compared to a no-horse contact active control. The objective of this study was to examine whether significant improvements of irritability, hyperactivity, social, and communication behaviors observed in participants randomized to receive a 10-week manual-based THR intervention were sustained 6 months after the intervention conclusion. Participants' caregivers from both the THR (*n* = 36) and active control (*n* = 28) groups completed a measure of irritability and hyperactivity behaviors (primary outcome variables). Additionally, only the THR group participants completed the full battery of study outcomes assessments. Between group comparisons examining the extended interval from baseline (1-month pre-intervention assessment) to 6-months after the intervention revealed that the THR group maintained reductions in irritability behavior at a 0.1 level (effect size = 0.32, *p* = 0.07). (Effect size = 0.32, *p* = 0.07), which was 73% of efficacy preserved from the primary post-intervention endpoint (within 1-month post-intervention). Hyperactivity behaviors did not sustain this same trend. Comparisons from baseline and 6-months after the intervention revealed that the THR group sustained significant initial improvements made in social and communication behaviors, along with number of words and different words spoken during a standard language sample. This is the first known study to examine and demonstrate the longer-term effects of THR for individuals with ASD and warrants a more thorough evaluation of whether the effects of THR are maintained for at least 6-months after the intervention compared to a control.

Clinical Trial Registration Information: Trial of Therapeutic Horseback Riding in Children and Adolescents with Autism Spectrum Disorder; http://clinicaltrials.gov; NCT02301195.

## Introduction

Along with the diagnostic social, communication, restricted, and repetitive behavior features of autism spectrum disorder (ASD) this population has particular difficulties with emotion regulation (1). Emotional dysregulation and related aberrant behavior responses (e.g., irritability and aggression) can detrimentally affect the daily social functioning of this population (2). Such issues can also contribute to an increase risk of exhibiting highly inappropriate and unsafe behaviors, the consequences of which can erode the quality of life (QoL) for the child with ASD and caregivers (3–5). The fact that there is no “one-size-fits- all” ASD intervention package (6) fuels a particular interest in seeking complementary and alternative ASD treatment options (7). One increasingly popular practice is the inclusion of animals in interventions to enhance human health, quality of life or well-being, known as animal-assisted intervention (AAI) (8). The use of AAI for individuals with ASD has been hypothesized to provide a unique social partnership experience with the animal, one that can reduce arousal levels (i.e., dampen stressed/anxious states) and can address the unique social, communication, and behavior challenges of individuals with ASD (9).

Emerging evidence for the benefits of animals on the health and well-being of individuals with ASD is highlighted by recent systematic literature reviews (10, 11). Research on AAI for ASD has increased in recent years, from only 14 studies meeting inclusion criteria for empirical research between 1989 and 2012, to 28 studies between 2012 and 2015. Early studies reported improvements in social and communication skills, decreases in ASD symptom severity, amelioration of behavior problems (e.g., aggression), reduced stress, and enhanced quality of life (10). However, a majority of these studies lacked methodological rigor, making these findings difficult to interpret or rely upon. Although diverse methods continue to be employed methodological quality of some of the more recent AAI studies have improved (11). In this more recent review of 22 out of 28 AAI studies, social interaction skills was identified as the most consistent outcome reported with additional outcome indications of improved communication skills, positive emotions, and reduced arousal levels (11). In this same review, equines were the most common animal species included in AAI (55% of studies) (11).

A systematic mapping review of equine-assisted activities and interventions (EAAT) studies with the ASD population revealed a wide variety of intervention methods ranging from equine assisted activities (EAA) (e.g., psychoeducational horseback riding, therapeutic riding) involving riding instructors, coaches or trainers, and equine-assisted therapies (EAT) (e.g., hippotherapy, simulated developmental horse-riding) involving therapists (e.g., occupational or physical therapists) and therapeutic riding instructors (12). In 31 of 33 studies reviewed, riding the horse was a key component, but EAA and EAT had different aims. Horsemanship, communication, and social skills were an emphasis of EAAs, with 13 out of 25 involving group sessions. Conversely, the eight EAT studies did not always specify group or individual session type, instead focused on the use of the horse's movement to target physical and sensorimotor functioning. Outcome improvement areas reported by EAA studies included social interactions, communications, sensory processing, movement control, ASD symptom severity, and QoL, whereas EAT studies reported outcome improvements in motor control and adaptive living skills (12).

Although THR appears to be a wide-spread practice that has become popular for individuals with ASD, few studies have systematically validated the effects of THR for individuals with ASD following recommended guidelines for ASD research of therapeutic interventions (13). Such a practice is necessary to guide consumers' AAI treatment choice making and third-party payers' interest in funding evidenced-based AAI. In response to this need, this research team conducted the first known large-scale (*N* = 127) randomized controlled trial (RCT) of THR compared to a no-horse activity control with children ages 6–16 years diagnosed with ASD (14). Results showed a significant medium effect size improvement in participants from the THR group (*n* = 58) compared to the control (*n* = 58) on measures of irritability, hyperactivity, social cognition, social communication, and total words and new words spoken during a standardized language sample.

There is a paucity of research examining the longer-term maintenance of AAI benefits for individuals with ASD beyond immediate outcomes. A recent follow-up study by Hall et al. (15) examined the maintenance of the immediate observed improvements in family functioning and stress made after families of children diagnosed with ASD either acquired a dog or did not (16, 17). This two-and-a-half-year follow-up revealed maintenance of family functioning gains in the subset of those who followed-up from the intervention group (*n* = 22) compared to the control (*n* = 15) (15). One study to date has attempted to prospectively examine residual effects of THR for children with ASD (18). That study conducted a repeated measure, interrupted treatment design to evaluate 21 participants with ASD before and after 10-weeks of THR. An unintentional 6-week break from treatment interrupted the study design. Participant outcome assessment following this initial 16 week-period indicated that participants showed improvements in sensory processing abilities and autism-related symptoms. However, none of these improvements remained when re-evaluating participants after discontinuing THR for 6 weeks. After re-introducing 6 weeks of THR, an immediate follow-up assessment revealed initial improvements resumed. However, of note, this study had several methodological limitations including only teacher-report that limited outcome evaluation to the school environment, lack of a control condition, and an unplanned 6-week break during the initial treatment phase (18). Examining the durability of AAI improvements is an important area of research needed in the growing effort to validate the efficacy of these interventions.

This article presents 6-month follow-up data from a subset of participants who were randomized as part of a previously published RCT to receive either 10-weeks of THR intervention or 10-weeks of a barn activity (BA) control group with no exposure to horses (14). In this study, we examined whether the behavioral, social, and communication improvements remained 6 months after the completion of the THR intervention.

## Materials and methods

The following summarizes the methods of our previously published RCT (14), which was conducted at a Première certified PATH international center follows industry guidelines to insure horse welfare. For additional details concerning participant consent/assent process, inclusion, and exclusion criteria, ASD study diagnostic confirmation, screening, randomization, measures, and interventions, please see the discussion in Gabriels et al. (14).

### Participants

At the onset of the RCT (14), all of the 127 participants ages 6–16 years with a study confirmed diagnosis of ASD were invited to engage in this 6-month follow-up assessment, approved by the Institutional Review Board (IRB) at the first author's institution. Specifically, participants and caregivers completed an informed consent/assent process, giving consent for three evaluation points: (1) baseline assessment, (2) post-intervention assessment, and a (3) 6-month post-intervention follow-up assessment contingent on agreement to refrain from continuing participation in THR for 6-months after the initial intervention phase. The informed consent process included mention of monetary incentives offered for each assessment period of the study, including the 6-month follow-up. From the 127 participants enrolled in the RCT (14), only 116 participants were eligible to complete this third study phase because they had completed RCT baseline (pre-intervention) assessments. However, only a subset of these 116 participants, 96 were invited to be included in the follow-up assessment process, because they completed 1-month post-intervention follow-up assessments and chose to refrain from continued participation in THR until after the 6-month follow-up evaluation period. Six months after completing the intervention phase of the RCT (14), the study coordinator contacted these 96 participants. A subset of these 96 participants contacted (64/66.67%) responded (THR group *n* = 36; control group *n* = 28). Of the 32 participants who did not complete this third study phase, two were no longer living with the same caregivers, rather were living in community-based placements, and the remainder were otherwise unable to schedule and/or complete study forms. Participating families received compensation for travel to study visits and for completing and returning the study questionnaire.

### Study design

Following institutional review board-approved informed consent/assent procedures for the RCT (14), participants were randomized in a 1:1 ratio to receive either a 10-week THR group or a no-horse barn activity (BA) control group based on a priori randomization list generated study statistician. This was stratified by participants' nonverbal intelligence quotient (NVIQ) standard score (85 or >85) measured by the Leiter-R (19). Weekly intervention and control group lessons lasted 45-min, consisted of two to four participants, were led by a THR instructor, followed a consistent routine, and were taught horsemanship skills via activities tailored to ASD learning styles outlined in the THR intervention manual (20). Specifically, the THR group participants each had one or multiple assigned volunteers (one horse leader and up to two side walkers) and the BA group participants each had one assigned volunteer. BA group participants had no contact with horses at the riding center, but were just able to view horses from a distance. There was a life-sized stuffed horse present in the BA group for hands-on learning related to the weekly horsemanship topic.

### Six-month outcome measures

Six months following the completion of the intervention phase of the study, the same caregiver who completed baseline (pre-intervention) study forms for THR (*n* = 36) and control (*n* = 28) groups, completed the 6-month follow-up study measures. Only the THR group participants (*n* = 36) were scheduled to come into the study clinic site for a re-administration of all baseline assessment measures (i.e., language assessment and social-communication caregiver report form). The speech therapist, who had conducted the baseline and post-intervention evaluations as a blinded evaluator, was unblinded at the 6-month follow-up, because only THR participants received the 6-month follow-up language assessment. However, baseline and post-intervention assessments were not available to the speech therapist during the 6-month evaluation period.

The Irritability and Hyperactivity subscales of the Aberrant Behavior Checklist-Community (ABC-C) (21) were the primary outcome measures for the RCT (14). For this follow-up, the subset of caregivers from both groups (THR and BA) were asked to complete the ABC-C (21). The ABC-C is a 58-item symptom checklist that assesses problem behaviors /self-regulation in children and adults with developmental disabilities (22, 23) and is commonly used as a primary outcome measure in psychopharmacological studies with the ASD population (21). Caregivers received this questionnaire electronically or by mail and were asked to report on participants' current (within the past 4 weeks) observed irritability and hyperactivity behaviors.

The persistence of the previously-reported (14) improvements in social communication and social-cognition behaviors observed in the THR group, were again measured using the Social Responsiveness Scale (SRS) (24) completed by the subset of caregivers from the participants who completed the THR intervention. The SRS has high internal consistency and retest temporal stability in males and females with ASD (25).

To evaluate the persistence of word fluency improvements made in the THR group compared to the control from baseline to post-intervention (14), the subset of participants from the THR group were again administered the Systematic Analysis of Language Transcripts (SALT) (26) 6-months post- the THR intervention. The SALT (26) consists of a 5-min standardized language sample that includes software to structure the collection, transcription, and analysis of language samples obtained from individuals, including those with ASD.

### Statistical analysis

SAS 9.4 was used for all the analyses (SAS Institute Inc.,)^1^. Participant characteristics and outcome data were compared between the THR and BA groups using two-sample *t*-test or chi square test as appropriate. The outcome analysis used a linear mixed effects model (LMM). For irritability and hyperactivity, the fixed effects of LMM included the classification variables of evaluation time (i.e., baseline, post-THR, and 6-month follow-up) and group indicator (THR or BA) as well as their interaction. Statistical test of the interaction of group by time was used to examine the significance of efficacy. Cohen's D effect size for efficacy is estimated based on this interaction test using 2 times *t* value divided by degree of freedom. Other outcome variables collected only for THR participants were also analyzed by LMM. Compound symmetry was the covariance structure used for all LMM analyses.

## Results

### Patient demographics and baseline clinical data

Summary statistics of demographic and baseline clinical characteristics for the 64 participants who chose to follow-up 6-months post the initial THR intervention (*n* = 64) and those participants (*n* = 52) who did not follow-up are listed in Table 1. There were no statistically significant differences between these two groups with respect to demographic and clinical data collected, except for the fact that those participants who did not chose to follow-up tended to travel from farther distances to the riding center (see Table 1).

**Table 1:** Demographics for participants with follow-up at 6 months' post-treatment and those without follow-up.

**Characteristic**	**With 6 months follow-up**				**Without 6 months follow-up**			
	**THR**	**Barn**	**Total**	***p*** ^a^	**THR**	***p*** ^b^	**Barn**	***p*** ^b^
No. of participants	36	28	64		22		30	
Age, [Mean (SD)], years	10.7 (2.9)	9.4 (2.5)	10.1 (2.8)	0.09^*^	10.3 (3.7)	0.67	10.5 (2.8)	0.14
Gender, males/females (counts)	29/7	25/3	54/10	0.34	20/2	0.46	27/3	1.0
IQ [Mean (SD)]	88.4 (25.1)	89.2 (19.8)	88.8(22.8)	0.89	83.8 (26.4)	0.51	83.1 (25.2)	0.31
Repetitive behavior scale total score [Mean (SD)]	38.1 (22.4)	37.2 (19.8)	37.7 (21.0)	0.86	37.7 (18.7)	0.94	38.9 (20.1)	0.74
Community psychiatric diagnoses, Y/N(Counts)	18/18	11/17	29/35	0.45	10/12	0.79	17/13	0.20
Mood disorder, Y/N(Counts)	6/30	5/23	11/53	1.0	2/20	0.70	7/23	0.75
Anxiety disorder, Y/N(Counts)	10/26	2/26	12/52	0.05^*^	6/16	1.0	7/23	0.15
ADHD, Y/N(Counts)	10/26	8/20	18/46	1.0	7/15	0.78	8/22	1.0
Learning disability, Y/N(Counts)	3/33	8/20	11/53	0.25	0/22	0.28	1/29	1.0
Current seizure disorder, Y/N(Counts)	1/35	0/28	1/63	1.0	0/22	1.0	2/28	0.49
Psychotropic medicine, Y/N(Counts)	17/19	11/17	18/36	0.53	10/12	1.0	18/12	0.19
Distance traveled to riding center [mean (SD)]	28.3 (6.4)	22/0 (5/4)	25.5 (19.3)	0.20	34.92(19.5)	0.20	31.6 (16.6)	0.049^**^
Latino/Hispanic, Y/N(Counts)	5/31	5/23	10/54	0.66	5/17	0.48	6/23	1.0
Race (counts)				0.24		0.38		0.90
American Indian or Alaska native	0	2	2		0		1	
Asian/ Hawaiian/Pacific Islander	0	2	2		2		1	
Black or African American	1	0	1		0		0	
White	30	20	50		18		25	
Mixed Race	4	1	5		1		1	
Other	1	2	3		0		2	
Missing	0	1	2		1		0	

a p-value for comparisons between THR and Barn group among participants with 6-month follow-up.

b p-values for comparisons between participants with 6-month follow-up and those without respectively for THR and Barn participants.

** p < 0.05;

* *p < 0.1 (Significant p-values are in bold)*.

### Between group efficacy maintenance at six-month follow-up

**Figure 1:**
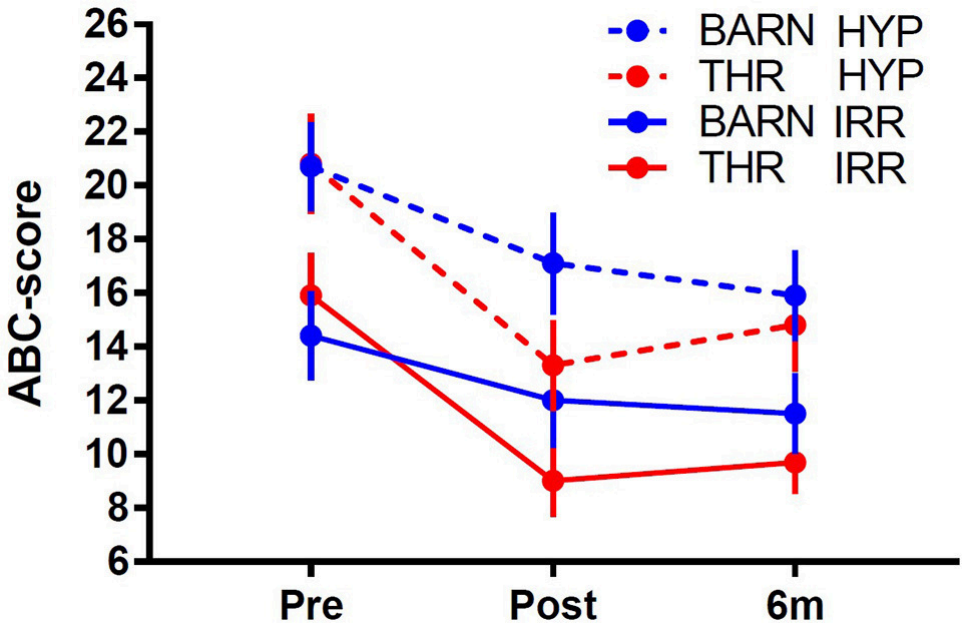
Profile of irritability (IRR) and hyperactivity (HYP) over three assessment periods. Note, the typical clinical threshold for the ABC-C irritability subscale is >14–16 in psychopharmacology clinical trials for the ASD population [e.g., (27, 28)].

For the subgroup of participants from the RCT (14) who completed the 6-month follow up assessment (THR group, *N* = 36; BA group, *N* = 28), the THR group experienced significantly more improvements (effect size = 0.44, *p* = 0.016) on the ABC-C (21) Irritability subscale between pre-intervention and within 1-month post-intervention as compared to the BA group (Table 2 and Figure 1). This efficacy is consistent with the larger RCT study results (14). Examining the extended interval from baseline to 6-months after intervention, showed significance at the 0.1 level (effect size = 0.32, *p* = 0.07) results favoring the THR group for the ABC-C (21) Irritability subscale. The observed effect size at 6 months are 73% of that at post-intervention in this sample and 64% of that observed in the larger RCT study. For the ABC-C (21) Hyperactivity subscale, the THR group showed a greater (non-significant) improvement compared to the BA group from pre-intervention to within 1 month post-intervention (effect size = 0.32, *p* = 0.08), in contrast to the significant finding in the original RCT study (14). There was no significant difference; however, when examining the extended interval from baseline to 6 months after intervention for the hyperactivity subscale (effect size = 0.09, *p* = 0.61), indicating efficacy of THR on hyperactivity was not sustained.

**Table 2:** Results of repeated measures analysis of variance (*N* = 36 for THR and *N* = 28 for Barn group)^a^.

**Outcome**	**Group**	**Baseline**	**EOT**	**6 Months post Intervention**	**Baseline minus EOT**	**ES** ^b^	***p***
Irritability	Barn	14.43 (8.69)	11.96 (9.29)	11.50 (7.87)	2.46 (1.10)		**0.03** ^**^	2.93 (1.10)		**0.01**	0.46 (1.10)	0.67
	THR	15.86 (9.52)	9.00 (8.08)	9.69 (6.87)	6.86 (1.42)		<**0.001** ^**^	6.17 (1.42)		<**0.001**	−0.69 (1.42)	0.63
	Difference	−1.43 (2.13)	2.96 (2.13)	1.81 (2.13)	−4.40 (1.79)	0.44	**0.02**	−3.24 (1.79)	0.32	0.07	1.16 (1.79)	0.52
Hyperactivity	Barn	20.71 (20.75)	17.07 (13.28)	15.86 (14.81)	3.64 (1.59)		**0.02**	4.86 (1.59)		**0.003**	1.21 (1.59)	0.45
	THR	20.75 (20.71)	13.28 (17.07)	14.81 (15.86)	7.47 (1.43)		<**0.001**	5.94 (1.43)		<**0.001**	−1.53 (1.43)	0.29
	Difference	−0.04 (2.48)	3.79 (2.48)	1.05 (2.48)	−3.83 (2.14)	0.32	0.08	−1.09 (2.14)	0.09	0.61	2.74 (2.14)	0.20

a Sample means and standard deviation (SD) were reported for Baseline, end of 10-week treatment (EOT) and 6 month intervention follow-up. Mean and standard errors for change between time points and between-group difference (Baseline minus EOT, Baseline minus 6 months, & EOT minus 6 months). P-values for the overall test of time by group interaction were 0.0431 for irritability and 0.1868 for hyperactivity.

b Cohen's D Effect size for efficacy at EoT or 6 months, estimated by 2 times t value divided by sqrt (DF).

** p < 0.05;

* *p < 0.1*.

### Six-month follow-up of the THR group

**Figure 2:**
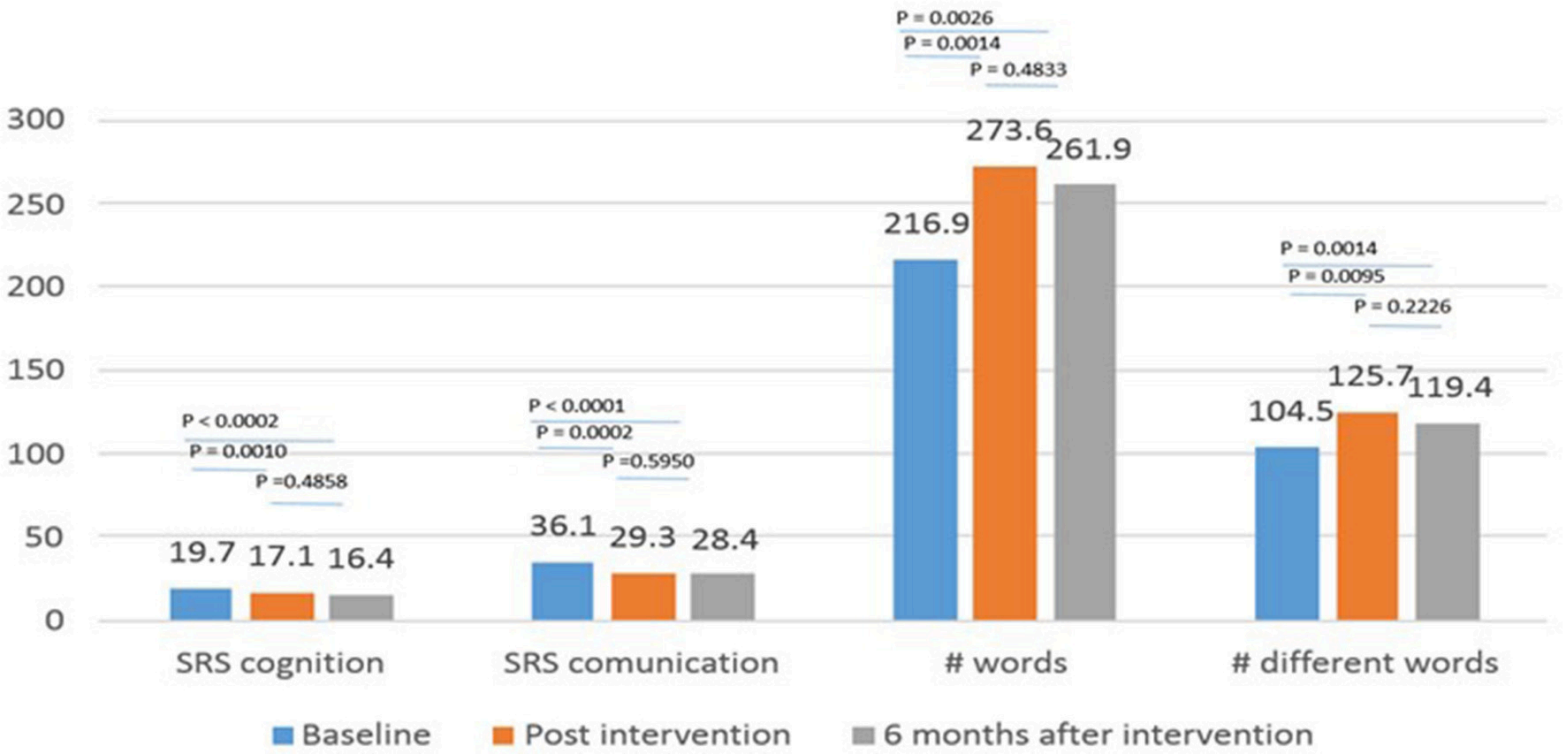
Mean scores at baseline, post-intervention and 6 months after THR.

Consistent with the original RCT study (14), significant improvements were observed in the THR group participants who completed the 6-month follow-up data collection using the SRS (24) Social Communication and Social Cognition subscales along with number of words and different words spoken during the SALT (26) from baseline (within 1-month pre-intervention) to 1-month post-intervention (each *p* < 0.01). These post-intervention changes sustained from 1-month post-intervention to the 6-month follow-up period (see Figure 2). Specifically, there was significant improvement for each outcome (*p* < 0.01) between baseline and 6 months' post-intervention, while there was not a significant difference between 1-month post-intervention and 6 months' post-intervention.

## Discussion

This report presents follow-up data from a subset of participants (*n* = 64) in a previously published RCT (14) study of the effects of a 10-week THR group intervention on children and adolescents with an ASD compared to a no-horse contact activity control group. Results from this follow-up study show that in the subset of THR participants measured, they retained some of their initial improvements made in irritability compared to the BA control. Additionally, an exploratory analysis of only the THR group revealed that they sustained their significant initial improvements made in social and communication behaviors, along with number of words and different words spoken during a standard language sample for at least 6 months following the completion of the THR intervention.

For reference, the previously published results of that RCT reported that after 10 weeks of intervention (THR or BA control), participants in the THR group showed significantly more improvements on the in irritability and hyperactivity behaviors compared to the BA control (14). The THR group also showed significant improvements on the SRS (24) subscales of social cognition and communication and used a greater number of different words as measured by the SALT (26), while the control group did not show similar improvements (14). The significant irritability and hyperactivity effects began by the fifth week of the THR intervention. Although the BA control showed significant within-group improvements in irritability and hyperactivity behaviors at end of intervention and 6 months after intervention, such improvements could be due to a variety of factors other than the BA group (e.g., placebo response). It would be misleading to make conclusions about the effects of this active (BA) control intervention due to the absence of a nonintervention control for comparison, a necessary element to test treatment effects as discussed in the literature [e.g., (29, 30)].

These results suggest that THR may be an effective complementary intervention to enhance social and verbal core symptoms of ASD, and to reduce irritability behaviors. Given the results of this study, particularly the lingering effects on irritability behaviors, we hypothesize that our THR manual-based approach may induce a reduction in arousal states, dampening stress/anxiety, in youth with ASD. Therefore, an important next step might be to examine the physiological regulation mechanisms involved in THR may explain (at least partially) improved outcomes in youth with ASD. Additionally, this study's finding of the long-term sustained improvements in irritability behaviors have clinical practice implications for youth with ASD that can add to the current standard of practice of administering anti-psychotic medications (i.e., Risperidone and aripiprazole) to reduce symptoms of irritability in this population (ages 5–16 years and 6–17 years) (31–33). For example, it has been proposed that THR might be a safe adjunct intervention to facilitate lowering medication dosages in this population (34). Finally, this study expands the AAI research base by prospectively examining the residual effects of THR for children with ASD.

The limitations of this study include its small sample size due to the high dropout rate 6 months following the initial intervention phase. This limits the validity of the results, as findings may not represent the greater population of youth with ASD. Another limitation is the fact that for those participants who did not chose to follow-up, attrition might be because they lived farther away from the study site. This also presents a possible selection bias. Even though we only included participants who refrained from engaging in THR for 6-months following the initial intervention, we did not specifically assess what other, if any, contact with horses was made during that time, which is a limitation. Another limitation is the fact that this study did not assess the efficacy of THR compared to the BA control group on all outcome measures employed in the previously published RCT (14). This limits the validity of these study results and warrants a more thorough evaluation of whether the positive effects of THR can be maintained for at least 6-months after the THR intervention compared to a control.

Despite these limitations, this study provides useful preliminary data to both support and extend the significant findings from our previously published pilot study and RCT (14, 35). This study also provides suggestions for future investigations of the longer-term benefits of THR in children and adolescents with ASD. For example, future investigations should consider the addition of incentives to lower follow-up attrition rates such as conducting outcome evaluations in closer proximity to participants' residence and providing increased monetary incentives for completing follow-up assessments. Future THR studies may consider collecting follow-up outcome assessments at several time points post-intervention to elucidate information regarding the lingering time course of outcome improvements. Finally, additional long-term outcome research will help to establish empirical evidence for THR as a valid intervention for youth with ASD, one that leads to the acquisition and long-term maintenance of behaviors skills that may enhance the quality of life for individuals with ASD and their caregivers.

## Author contributions

RG was the principal investigator of this study and contributed to writing the majority of this manuscript. ZP served as the statistical expert for this study and wrote the result section of this manuscript. GM served as a consultant for this study and provided editorial edits to this manuscript. BD served as the clinical coordinator for this study and provided editorial edits to this manuscript. NG assisted in writing the introduction of this manuscript.

### Conflict of interest statement

RG is a co-author of the book, Growing Up with Autism: Working with School-aged Children and Adolescents (Guilford Press) and the book, Autism from Research to Individualized Practice (Jessica Kingsley Publishers), from which she receives royalties. Current grant funding for RG provided by Simons and Lurie Foundations and The Human-Animal Bond Research Institute (HABRI) Foundation. The remaining authors declare that the research was conducted in the absence of any commercial or financial relationships that could be construed as a potential conflict of interest.

## Footnotes

^1^SAS Institute Inc. “SAS” (Cary, NC).
